# A Unified Control Framework for Self-Balancing Robots: Addressing Model Variations in Wheel-Legged Platforms and Human-Carrying Wheelchairs

**DOI:** 10.3390/s25237144

**Published:** 2025-11-22

**Authors:** Guiyang Xin, Boyu Jin, Chen Liu, Mian Jiang

**Affiliations:** 1 State Key Laboratory of Robotics and Systems, Harbin Institute of Technology, Harbin 150001, China; guiyang.xin@dlut.edu.cn; 2 School of Biomedical Engineering, Dalian University of Technology, Dalian 116024, China; 3 College of Future Technology, The Hong Kong University of Science and Technology (Guangzhou), Guangzhou 511400, China; 4 School of Marine Science and Technology, Northwestern Polytechnical University, Xi’an 710072, China; 5 School of Mechatronic Engineering and Automation, Foshan University, Foshan 528225, China

**Keywords:** self-balancing robots, wheel-legged robots, LQR

## Abstract

Self-balancing robots, with their compact size, are capable of achieving high agility. Small wheel-legged self-balancing robots have demonstrated significant potential across various applications. However, expanding small self-balancing robots to larger sizes to serve as personal transport tools is a more attractive and impactful direction than further miniaturization or confinement to niche laboratory demonstrations. This paper presents the development of a small self-balancing robot, which is then scaled up to a larger version designed to carry human passengers as a self-balancing wheelchair. A unified control framework, built around a shared core of online model-updating LQR for balance and PD for steering, is applied to both robots. This core is supplemented with platform-specific modules, such as a dedicated leg controller for the wheel-legged robot, to handle distinct dynamic maneuvers. The LQR controller is implemented for balance control in both robots. Additionally, a dedicated leg controller is applied exclusively to the small wheel-legged robot to enable dynamic maneuvers, such as jumping. A series of experiments conducted with the final prototypes validate the effectiveness of the control systems and highlight the robots’ application potential.

## 1. Introduction

The concept of self-balancing robots has gained considerable attention in both research and industry due to their versatility in various applications, ranging from personal mobility devices to delivery systems and assistive robots. Notable examples include two-wheeled robots (e.g., Segway [1] and JOE [2]) and wheel-legged robots (e.g., Ascento [3] and Handle [4]). While self-balancing robots offer significant agility, it is important to delineate the application domains where they hold a distinct advantage over statically stable alternatives, such as four-wheeled or tracked robots. The primary strengths of self-balancing platforms lie in their compact footprint, zero turning radius, and dynamic obstacle-crossing capabilities. These traits make them ideally suited for operations in tight, human-populated environments like crowded offices, factory floors with narrow aisles, or even the interior of aircraft and trains, where the larger turning circle of a conventional robot would be impractical. From a theoretical perspective, self-balancing systems are interesting platforms because they are nonlinear, underactuated, and inherently unstable [5,6,7,8]. These characteristics make them challenging to control, especially when subjected to external disturbances or model variations.

In this study, we developed two types of self-balancing robots: one is a conventional, small-sized wheel-legged robot, and the other is a larger self-balancing robot capable of carrying a person. The primary challenge in controlling the wheel-legged robot lies in the changing leg dynamics, which result in variations in the system model. In contrast, the challenge in the self-balancing wheelchair is the variation in system equilibrium caused by the unknown weight and center of mass (CoM) of the passenger.

### 1.1. Related Works

Without model variation, there are mature solutions for the control of self-balancing robots. A backstepping controller dedicated to balance control and a PID controller for velocity tracking were combined to control a two-wheeled self-balancing robot in [9]. To overcome external disturbances, researchers have proposed various optimization methods for tuning PID gains, such as artificial bee colony optimization, particle swarm optimization, grey wolf optimization, and the cuckoo search algorithm [10,11]. However, PID controllers with fixed gains are not ideal for nonlinear systems like self-balancing robots. As a result, adaptive fuzzy PID control [12,13] and neural network-based PID control [14] have been developed to adjust PID gains online, improving system performance under varying conditions.

Model-based controllers, such as LQR and MPC, have demonstrated greater robustness compared to PID controllers [15], particularly for balance control. Self-balancing robots are often modeled as inverted pendulums [2,16], which can maintain an upright position and stable movement despite their inherent instability. However, the mobile inverted pendulum model does not account for wheel inertia. Neglecting wheel inertia can lead to an underestimation of the system’s overall inertia, resulting in a controller that is too aggressive and performs poorly in practice. The wheeled inverted pendulum (WIP) model [17,18] provides a more accurate dynamic representation. While LQR controllers improve robustness, their reliance on linearization around the equilibrium state limits their effectiveness to small pitch angles. To address this limitation, nonlinear MPC controllers [15,19,20,21,22] have been proposed for extreme dynamic maneuvers. However, these methods are computationally intensive and challenging to implement in real-time, especially when the system model changes dynamically.

Recent advancements in adaptive control and reinforcement learning have also shown promise for self-balancing robots. For instance, ref. [23] proposed an adaptive zero-offset angle identification algorithm to compensate for deviations caused by changes to the robot’s center of gravity, while ref. [24] applied deep reinforcement learning to optimize control policies for wheel-legged robots in unstructured environments. Additionally, ref. [25] introduced a disturbance observer-based approach to enhance the robustness of wheeled inverted pendulum systems, and ref. [26] provided a comprehensive review of adaptive control techniques for underactuated balance robots. These approaches highlight the growing interest in combining traditional control methods with modern machine learning techniques to enhance system performance [27,28].

### 1.2. Overview

**Figure 1:**
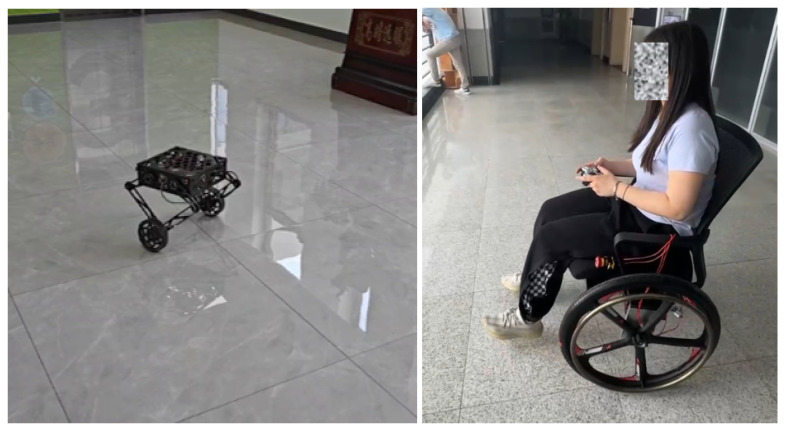
A small wheel-legged self-balancing robot and a large self-balancing wheelchair. See supplementary video [29] for dynamic maneuvers.

In this paper, we propose a unified control framework to address the model variation problem in wheel-legged self-balancing robots and human-carrying self-balancing wheelchairs. The two custom-built self-balancing robots are shown in Figure 1. The small robot employs a five-bar linkage mechanism as the leg, acting as an active suspension system, enabling the robot to overcome common indoor and urban obstacles such as thresholds, curbs, and uneven pavement; traverse uneven terrain; and even jump. The self-balancing wheelchair utilizes a force/torque sensor to estimate the person’s mass and center of mass (CoM). The dynamic models of both robots can be updated in real time. An LQR controller, with online computation, is employed to maintain balance based on the updated dynamic models, while the rotation speed is controlled by a PD controller. Crucially, this same LQR controller also regulates the robot’s forward/backward translational speed by tracking a desired velocity command, leveraging its inherent ability to control the entire sagittal-plane state vector. The proposed system is validated through extensive tests, demonstrating its ability to maintain balance, navigate obstacles, and perform dynamic maneuvers such as jumping. The main contributions of the paper are listed as follows.

1. A model updating method with an online computing LQR controller is proposed to solve the model changing problem for both robots, demonstrating versatility across platforms. We integrate force/torque sensor feedback for CoM estimation, enabling automatic recalibration of the equilibrium point. This is critical for passenger-carrying applications, where traditional LQR fails due to unmodeled CoM offsets.
2. Human-carrying, jumping, and obstacle-crossing experiments were conducted to demonstrate the real-world application potential of both robots.

The remainder of this paper is organized as follows: Section 2 presents the decoupled dynamic models of the self-balancing robots. The control framework and model update method are described in Section 3. Experimental results and analysis are provided in Section 4. Finally, the conclusions are presented in Section 5.

## 2. Problem Modification

The dominant part of a self-balancing robot system lies in the sagittal plane, which is responsible for balance control and translational motion. We will present the sagittal plane dynamics in Section 2.1. The rotation dynamics are ignored since a PD controller is used to control the turning motion. The wheel-legged sefl-balancing robot has a five-bar linkage closed-loop mechanism as the leg mechanism. We present the kinematics and statics of the leg mechanism in Section 2.2.

### 2.1. Sagittal Plane Dynamics

**Figure 2:**
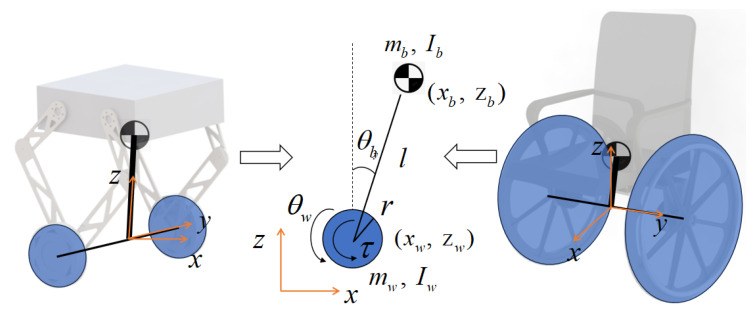
Two-dimensional mobile wheeled inverted pendulum system model projected on the sagittal plane (x-z plane).

Self-balancing robots are usually formulated as mobile wheeled inverted pendulum models or cart–pole systems for balance control. However, the cart–pole model cannot include the wheel inertia in the dynamics. In this work, we choose to use the mobile wheeled inverted pendulum model to derive the system dynamics. The two robots can be projected on their sagittal planes, as shown in Figure 2, where the WIP model is a 2D model. The derived WIP model is standard and aligns with the established dynamics in the literature, such as [17]. $\theta_{w}$ and $\theta_{b}$ are the wheel’s rotation angle and the inclination angle of the body, respectively. Here, $\theta_{w}$ is a continuous rotation angle, not restricted to a principal value. $m_{b}$, $I_{b}$, $m_{w}$ and $I_{w}$ are the masses and inertias of the body and the wheel of the 2D WIP model. *l* denotes the length between the wheel axle and the CoM of the body. *r* is the wheel radius. The coordinates of the wheel and body are denoted by $\left(x_{w},z_{w}\right)$ and $\left(x_{b},z_{b}\right)$, respectively. The positions of the system can be given by

(1) $$x_{b}=l\sin\theta_{b}+x_{w}$$

(2) $$z_{b}=l\cos\theta_{b}$$

(3) $$x_{w}=r\theta_{w}$$

The Lagrange’s equation of motion is used to derive the dynamics of the system. The kinetics and potential energy are computed as follows:

(4) $$V_{b}=0.5m_{b}\left({{\dot{x}}_{b}}^{2}+{{\dot{z}}_{b}}^{2}\right)+0.5I_{b}{{\dot{\theta }}_{b}}^{2}$$

(5) $$V_{w}=0.5m_{w}{{\dot{x}}_{w}}^{2}+0.5I_{w}{{\dot{\theta }}_{w}}^{2}$$

(6) $$P_{b}=m_{b}gl\cos\theta_{b}$$

Therefore, the Lagrange function L is given by

(7) $$L=V_{b}+V_{w}-P_{b}$$

The dynmaics of the WIP model can be represented by

(8) $$\frac{d}{dt}\left(\frac{\partial L}{\partial \dot{\theta_{b}}}\right)-\frac{\partial L}{\partial \theta_{b}}=-\tau$$

(9) $$\frac{d}{dt}\left(\frac{\partial L}{\partial \dot{\theta_{w}}}\right)-\frac{\partial L}{\partial \theta_{w}}=\tau$$

By substituting (1)–(3) into (8) and (9), the nonlinear dynamics of the WIP is as follows:

(10) $$M_{22}{\ddot{\theta }}_{b}=-M_{12}{\ddot{\theta }}_{w}\cos\theta_{b}+G\sin\theta_{b}-\tau$$

(11) $$M_{11}{\ddot{\theta }}_{w}=M_{12}\left(\sin\theta_{b}{\dot{\theta_{b}}}^{2}-\cos\theta_{b}{\ddot{\theta }}_{b}\right)+\tau$$

where parameters $M_{11}$, $M_{12}$, $M_{22}$, and *G* are

(12) $$\left\{\begin{array}{c} M_{11}=\left(m_{b}+m_{w}\right)r^{2}+I_{w}\\ M_{12}=m_{b}rl\\ M_{22}=m_{b}l^{2}+I_{b}\\ G=m_{b}gl \end{array}\right.$$

We define the state as $x={\left[\theta_{b}\;{\dot{\theta }}_{b}\;{\dot{\theta }}_{w}\right]}^{⊤}$. In order to rewrite the dynamics into a discrete-time state space description, we need to perform linearization at the equilibrium point $\theta_{b}=0$ by using the Taylor expansion with a time step Δt:

(13) $$x_{k+1}=Ax_{k}+B\tau_{k}$$

where $A=\left[\begin{array}{ccc} 1 & \Delta t & 0\\ \frac{M_{11}G\Delta t}{M_{11}M_{22}-M_{12}^{2}} & 1 & 0\\ -\frac{M_{12}G\Delta t}{M_{11}M_{22}-M_{12}^{2}} & 0 & 1 \end{array}\right]$ and $B=\left[\begin{array}{c} 0\\ -\frac{(M_{11}+M_{12})\Delta t}{M_{11}M_{22}-M_{12}^{2}}\\ \frac{(M_{22}-M_{12})\Delta t}{M_{11}M_{22}-M_{12}^{2}} \end{array}\right]$. It should be noted that *A* and *B* must be updated since *l* and $m_{b}$ can be changed when the leg length varies or a passenger sits on the wheelchair.

### 2.2. Leg Kinematics and Statics

**Figure 3:**
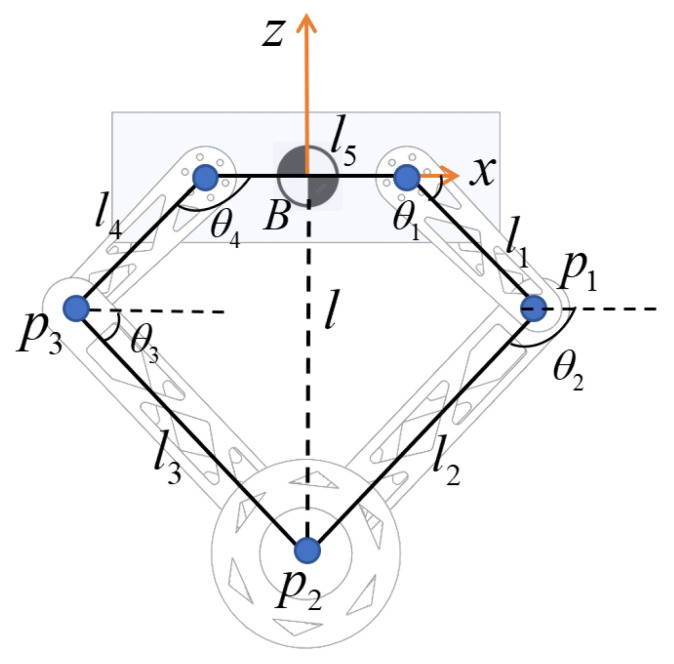
The five-bar linkage model of the robot’s leg.

Figure 3 shows the leg mechanism of the wheel-legged robot, which is a five-bar linkage close chain actuated by two motors. $\theta_{1}$ and $\theta_{4}$ are independently actuated by motors. While closed-form inverse kinematic solutions exist for the five-bar linkage, for our torque control application, we directly employ the Jacobian transpose for force control. Therefore, we derive the forward kinematics and statics to map the end-effector forces to the actuated joint torques.

Given $\theta_{1}$ and $\theta_{4}$, the positions of $p_{1}$ and $p_{3}$ are determined. Then, the position of $p_{2}$ in frame {B} can be expressed as

(14) $$\left\{\begin{array}{c} x_{p_{2}}=x_{p_{1}}+l_{2}\cos\theta_{2}=x_{p_{3}}+l_{3}\cos\theta_{3}\\ z_{p_{2}}=z_{p_{1}}+l_{2}\sin\theta_{2}=z_{p_{3}}+l_{3}\sin\theta_{3} \end{array}\right.$$

where $x_{p_{1}}=0.5l_{5}+l_{1}\cos\theta_{1}$, $z_{p_{1}}=l_{1}\sin\theta_{1}$, $x_{p_{3}}=-0.5l_{5}+l_{4}\cos\theta_{4}$, and $z_{p_{3}}=l_{4}\sin\theta_{4}$. By eliminating $\theta_{3}$, we have

(15) $$\theta_{2}=2\tan^{-1}\frac{B+\sqrt{A^{2}+B^{2}-C^{2}}}{A+C}$$

where $A=2l_{2}\left(x_{p_{1}}-x_{p_{3}}\right)$, $B=2l_{2}\left(z_{p_{1}}-z_{p_{3}}\right)$, and $C=l_{2}^{2}+{\left(x_{p_{1}}-x_{p_{3}}\right)}^{2}+{\left(z_{p_{1}}-z_{p_{3}}\right)}^{2}-l_{3}^{2}$.

Substituting (15) into (14) yields the coordinates of $p_{2}$. $\theta_{3}$ can be computed using the same method.

Taking the derivative with respect to time of (14) and eliminating ${\dot{\theta }}_{3}$, we obtain

(16) $${\dot{\theta }}_{2}=\frac{\left({\dot{x}}_{p_{3}}-{\dot{x}}_{p_{1}}\right)\cos\theta_{3}+\left({\dot{z}}_{p_{3}}-{\dot{z}}_{p_{1}}\right)\sin\theta_{3}}{l_{2}\sin\left(\theta_{3}-\theta_{2}\right)}$$

where ${\dot{x}}_{p_{1}}=-l_{1}{\dot{\theta }}_{1}\sin\theta_{1}$, ${\dot{z}}_{p_{1}}=l_{1}{\dot{\theta }}_{1}\cos\theta_{1}$, ${\dot{x}}_{p_{3}}=-l_{4}{\dot{\theta }}_{4}\sin\theta_{4}$, and ${\dot{z}}_{p_{3}}=l_{4}{\dot{\theta }}_{4}\cos\theta_{4}$. By substituting (16) into the derivative of (14), we can get the Jacobian matrix of the five-bar linkage mechanism:

(17) $$J=\left[\begin{array}{cc} \frac{l_{1}\sin\left(\theta_{1}-\theta_{2}\right)\sin\theta_{3}}{\sin(\theta_{2}-\theta_{3})} & \frac{l_{4}\sin\left(\theta_{3}-\theta_{4}\right)\sin\theta_{2}}{\sin(\theta_{2}-\theta_{3})}\\ -\frac{l_{1}\sin\left(\theta_{1}-\theta_{2}\right)\cos\theta_{3}}{\sin(\theta_{2}-\theta_{3})} & -\frac{l_{4}\sin\left(\theta_{3}-\theta_{4}\right)\cos\theta_{2}}{\sin(\theta_{2}-\theta_{3})} \end{array}\right]$$

Then, the statics of the five-bar linkage mechanism can be obtained:

(18) $$\tau_{\mathrm{leg}}=\left[\begin{array}{c} \tau_{1}\\ \tau_{4} \end{array}\right]=J^{⊤}\left[\begin{array}{c} F_{x}\\ F_{z} \end{array}\right]=J^{⊤}F$$

where $\tau_{1}$ and $\tau_{4}$ represent the output torques of the two motors that actuate the input links $l_{1}$ and $l_{4}$ of the five-bar linkage mechanism (see Figure 3). F is the virtual force vector applied at the foot-end (i.e., the wheel axle point $p_{2}$).

## 3. Control Framework

**Figure 4:**
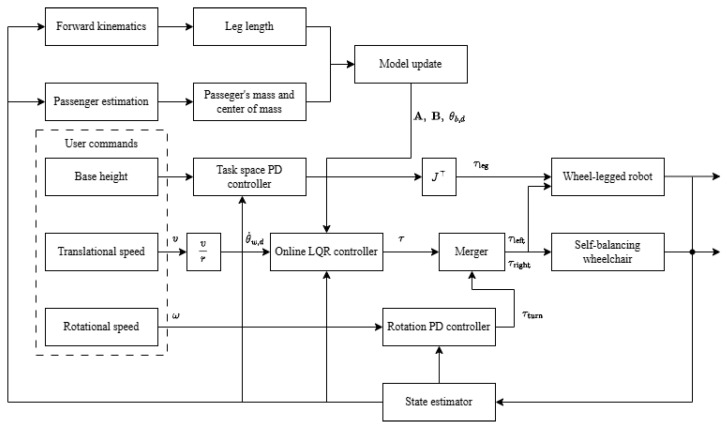
The five-bar linkage model of the robot’s leg. The framework consists of a universal control core and platform-specific control modules. The universal control core consists of an online model-updating LQR controller for balance and translational velocity tracking, while the wheel-legged robot has a task-space PD leg controller for height adjustment.

The control diagram for the robots is shown in Figure 4. The user commands include the steering translational speed *v*, rotational speed ω, and a height adjustment command which is unique to the wheel-legged robot. The LQR controller maintains balance while tracking the desired translational speed, whereas a PD controller regulates the rotational speed. An additional PD controller manages the base height for the wheel-legged robot. To account for variations in base height and the presence of an unknown passenger, the dynamic models are continuously updated by an algorithm.

### 3.1. LQR Controller

Since the linearized dynamic model, i.e., (13), has been derived, a LQR control problem can be formulated as follows:

(19) $$\begin{array}{cc} \min_{\tau_{0},\tau_{1},\ldots \tau_{\infty }}\; & \sum_{k=0}^{\infty }\left(x_{k}^{⊤}Qx_{k}+R\tau_{k}^{2}\right)\\ s.t.\; & x_{k+1}=Ax_{k}+B\tau_{k} \end{array}$$

where $Q\in R^{3\times 3}$ represent the weight matrices for the states, and $R\in R^{+}$ is a scalar for the control input. The solution of (19) is the well-known optimal feedback control law:

(20) $$\tau_{k}=-K\left(x_{k}-x_{d}\right)$$

where $x_{k}$ is the current state measured by the state estimator, $x_{d}$ is the desired state, and K is the optimal feedback control gain matrix.

(21) $$K={\left(R+B^{⊤}\mathrm{PB}\right)}^{-1}B^{⊤}\mathrm{PA}$$

where P is the unique positive definite solution to the discrete time algebraic Riccati equation:

(22) $$P=A^{⊤}\mathrm{PA}-A^{⊤}\mathrm{PB}{\left(R+B^{⊤}\mathrm{PB}\right)}^{-1}B^{⊤}\mathrm{PA}+Q$$

We solve the algebraic Riccati equation by iterating the dynamic Riccati equation of the finite-horizon case until it converges. This approach is termed the online LQR controller.

Note that the desired state $x_{d}={\left[\theta_{b,d}\;{\dot{\theta }}_{b,d}\;{\dot{\theta }}_{w,d}\right]}^{⊤}$ typically takes the form $x_{d}={\left[0\;0\;{\dot{\theta }}_{w,d}\right]}^{⊤}$ to maintain balance. However, the desired inclination angle $\theta_{b,d}$ is no longer zero if there is a horizontal bias in the passenger’s CoM relative to the robot’s CoM. The $\theta_{b,d}$, A, and B values will be updated in the following subsection.

### 3.2. Model Update Method

**Figure 5:**
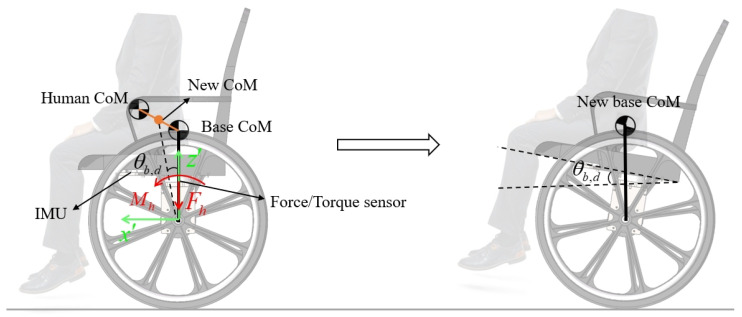
The illustration diagram for the wheelchair model update method.

The variation in leg length will cause a change in the CoM for the wheel-legged robot. It is easy to compute the new *l* and update A and B in (13). In contrast, the change in the model caused by the presence of a person in the wheelchair is more complex because the person’s mass and CoM are unknown. More importantly, the new CoM of the system will no longer align with the system’s centerline, as illustrated in Figure 5. The desired inclination angle $\theta_{b,d}$ must be changed since the IMU is fixed on the board. If we do not change the desired inclination angle $\theta_{b,d}$, the system will never settle down.

In our design, a force/torque sensor is used to connect the chair and the base of the wheelchair robot in order to estimate the person’s properties. As shown in Figure 5, we can compute the mass and the CoM bias based on the measured force and torques as follows:

(23) $$\left\{\begin{array}{c} m_{h}=F_{h}/g\\ x_{h}=M_{h}/\left(m_{h}g\right) \end{array}\right.$$

Assuming that each person’s CoM in height is the same as $z_{h}$, which is measured by the 3D CAD model, the new CoM can be calculated as follows:

(24) $$\left\{\begin{array}{c} x_{c}^{\prime }=\frac{{x_{h}}^{\prime }m_{h}}{m_{h}+m_{b}}\\ z_{c}^{\prime }=\frac{z_{h}^{\prime }m_{h}+z_{b}^{\prime }m_{b}}{m_{h}+m_{b}} \end{array}\right.$$

We can update the A and B values in (13) by substituting $m_{b}$ with $m_{b}+m_{h}$ and *l* with $l=\sqrt{{x_{c}^{\prime }}^{2}+{z_{c}^{\prime }}^{2}}$, respectively. The desired inclination angle $\theta_{b,d}$ also can be determined by

(25) $$\theta_{b,d}=\tan^{-1}\left(\frac{x_{c}^{\prime }}{z_{c}^{\prime }}\right)$$

Unlike traditional LQR controllers that use fixed model parameters, the equilibrium adjustment method enables our controller to handle unknown CoM offsets.

### 3.3. Sensitivity Analysis and Empirical Robustness

To rigorously analyze the stability of the proposed control framework, we examine the closed-loop dynamics under real-time model updates. The core of our control framework relies on the linearized discrete-time state-space model (13) of the self-balancing robot. It is a bounded-input bounded-output (BIBO) system since the system parameters ($m_{b}$, $m_{h}$, *l*, CoM) are physically bounded. The following theorem establishes the BIBO stability of the system when the LQR controller is updated online.

The proposed control framework involves a linearized model that is updated online, resulting in a time-varying system. A principled stability analysis for such a system would require a switched-linear or linear time-varying (LTV) framework, which is beyond the scope of this work. Instead, we provide a heuristic sensitivity analysis to offer insight into how parameter variations affect the control system.

**Theorem** **1** (BIBO Stability Under Model Updates)**.** *Consider the linearized system (13) with the LQR control law (20). If the model parameters $\left(l,m_{b}\right)$ are updated such that*

*1*. *The CoM deviation $\Delta l=|l_{k+1}-l_{k}|$ is bounded by $\Delta l_{\max}=\sqrt{\frac{\lambda_{\min}\left(Q\right)}{2∥P∥∥G∥}}$;*
*2*. *The mass variation $\Delta m_{b}=\left|m_{b,k+1}-m_{b,k}\right|$ satisfies $\Delta m_{b}<\frac{r^{2}I_{w}}{2l^{2}\left(m_{w}+m_{b}\right)}$*

*then the closed-loop system is BIBO stable, and the state $x_{k}$ converges to a neighborhood of $x_{d}$ with ultimate bound:*

(26) $$∥x_{k}-x_{d}∥\leq \frac{\epsilon }{\sigma_{\min}\left(Q\right)},$$

*where ϵ accounts for linearization errors and $\sigma_{\min}\left(Q\right)$ is the smallest singular value of Q.*

**Proof.**  Let $e_{k}=x_{k}-x_{d}$ be the tracking error. The Lyapunov candidate $V\left(e_{k}\right)=e_{k}^{⊤}Pe_{k}$ satisfies

(27) $$\Delta V=V\left(e_{k+1}\right)-V\left(e_{k}\right)\leq -e_{k}^{⊤}Qe_{k}+2∥P∥∥G∥\Delta l∥e_{k}{∥}^{2}.$$

For $\Delta l<\Delta l_{\max}$, ΔV<0 ensures convergence and stability. Mass variation bounds follow similarly from (12). □

**Lemma 1** (Positive Definiteness of **P**)**.** *The Riccati solution P remains positive definite if the length update rate satisfies*

(28) $$\left|\frac{\Delta l}{l}\right|<\frac{1}{2\kappa (A)},$$

*where κ(A) is the condition number of A.*

### 3.4. Turning Controller

Since the wheels are controlled by torque commands generated by the LQR controller, rotation speed tracking is directly controlled by a PD controller instead of using differential speed control.

(29) $$\tau_{\mathrm{turn}}=K_{p}^{\mathrm{turn}}\left(\omega_{d}-\omega \right)+K_{d}^{\mathrm{turn}}\left({\dot{\omega }}_{d}-\dot{\omega }\right)$$

where ${\dot{\omega }}_{d}=0$, $\omega_{d}$ is sent by the users, and ω and $\dot{\omega }$ are measured by the IMU.

The torque commands sent to the left and right wheels can be calculated as follows:

(30) $$\left\{\begin{array}{c} \tau_{\mathrm{left}}=\frac{1}{2}\left(\tau -\tau_{\mathrm{turn}}\right)\\ \tau_{\mathrm{right}}=\frac{1}{2}\left(\tau +\tau_{\mathrm{turn}}\right) \end{array}\right.$$

where τ is the result generated by the LQR controller, i.e., (20).

The torque split defined in Equation (31) introduces a potential coupling where the turning torque $\tau_{\mathrm{turn}}$ could disturb the sagittal-plane balance regulated by the LQR-derived torque τ. To justify the robustness of this decoupled design, we consider the timescale separation between the two control objectives.

The sagittal-plane dynamics of a self-balancing robot are inherently unstable, with a characteristic time constant on the order of a few hundred milliseconds. The LQR controller is therefore designed to be a high-bandwidth controller to stabilize this fast process. In contrast, the rotational dynamics of the robot are stable (behaving similarly to a damped rotational system), and the turning PD controller is tasked with tracking a user’s rotational velocity command, which is typically a low-frequency signal.

Due to this separation of timescales, the high-gain LQR controller can rapidly reject the low-frequency disturbances introduced into the sagittal plane by $\tau_{\mathrm{turn}}$. The turning controller’s output acts as a slow-varying disturbance that the balance loop effectively treats as a quasi-static load change, which it is designed to compensate for through its integral action (inherent in the state-feedback structure around a non-zero equilibrium). This rationale explains the empirical robustness observed in our experiments.

### 3.5. Leg Controller

Similarly to the turning controller, the leg positions relative to the robot’s base for the wheel-legged robot are also controlled using a PD controller.

(31) $$F=K_{p}^{\mathrm{leg}}\left(P_{3,d}-P_{3}\right)+K_{d}^{\mathrm{leg}}\left({\dot{P}}_{3,d}-{\dot{P}}_{3}\right)$$

where $P_{3,d}=\left[p_{3,d}^{x}\;p_{3,d}^{z}\right]⊤$ is the desired position of the wheel axle relative to the base, as shown in Figure 3, $p_{3,d}^{x}=0$, and $p_{3,d}^{z}$ is the given user command, which is important for jumping motion. A jumping trajectory generator is used to generate $p_{3,d}^{z}$. Then, the torque commands for the leg control motors will be computed by the statics (18). The purpose of the leg is to serve as an active suspension and maneuver generator, while the stability under the resulting dynamic loads is guaranteed by the unified LQR core.

## 4. Results

In this section, we evaluate the proposed control framework using velocity tracking, traversing steps, and human carrying experiments. The wheel-legged robot also shows its jump ability.

### 4.1. Experiments of the Wheel-Legged Robot

The wheel-legged robot is controlled by an onboard STM32H723 MCU (ST company, Geneva, Switzerland), which communicates with the six motors via a CAN bus. The six motors have the same peak torque limit, which is 7 Nm. A BMI088 IMU (Bosch company, Gerlingen, Germany) is connected to the MCU. The nominal length *l* is 0.2 m. The base mass is 2.4 kg, while the wheel mass is 0.7 kg. The radius of the wheel is 0.06 m. A Kalman filter is employed for optimal state estimation by fusing data from the IMU and wheel odometer.

For the wheel-legged robot, the high-level control loop runs on the STM32H723 MCU at 500 Hz, with a worst-case execution time of 1.5 ms, demonstrating the efficiency of the proposed algorithms even on an embedded platform.

#### 4.1.1. Velocity Tracking

**Figure 6:**
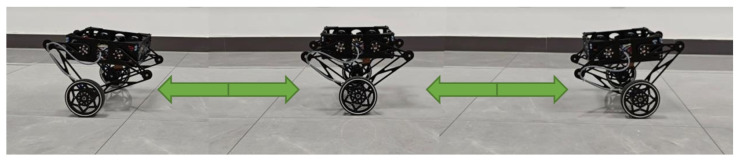
Snapshots of when the wheel-legged robot tracks a reciprocating motion.

**Figure 7:**
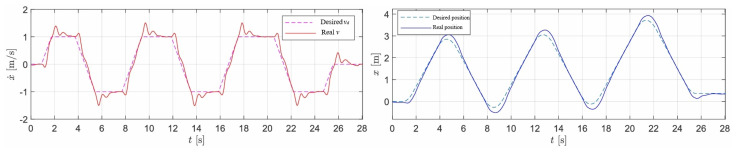
Velocity and position control performance along x axis.

**Figure 8:**
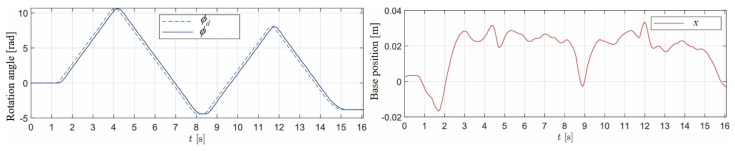
Rotation control performance.

In this experiment, the wheel-legged robot successfully tracked the desired translational and rotational velocities. Figure 6 presents snapshots of the robot during translational velocity tracking. Figure 7 illustrates the desired translational velocity along the *x*-axis and the corresponding desired position, which was derived by integrating the desired translational velocity. It is important to note that the proposed LQR controller was not designed to track the desired position. The comparison between the desired and measured positions is provided to offer readers an intuitive understanding of the reciprocating motion observed in this experiment. Figure 8 demonstrates the rotation control performance, where the desired rotational angle $\phi_{d}$ was generated by integrating the desired rotational velocity ω. The results indicate that rotational tracking achieved minimal errors, while the base position remained stable. The near-zero x-displacement in Figure 8 confirms excellent decoupling between rotational and translational motions. This experiment highlights the effective coordination between the LQR and the rotational PD controllers, where $K_{p}=30$ and $K_{d}=1$.

#### 4.1.2. Jumping

**Figure 9:**
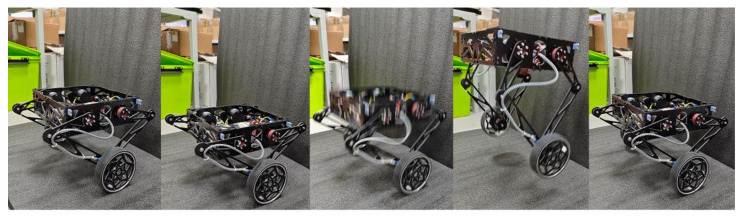
Snapshots when the wheel-legged robot is jumping.

**Figure 10:**
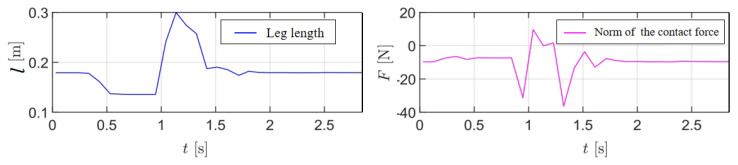
The leg length and contact force during the jumping motion.

In this experiment, the task space PD controller was evaluated through a jumping motion, as depicted in Figure 9. The desired end-effector trajectories were determined through iterative trials and then transformed into the base frame, with real-time leg length variations and ground contact forces recorded in Figure 10. The jumping sequence exhibited three distinct phases:

1. Preparation phase (0.3–0.9 s): The robot lowered its center of mass by retracting the legs, storing elastic energy in the system.
2. Takeoff phase (0.9–1.1 s): Rapid leg extension generated peak ground reaction forces of 38 N, propelling the robot vertically.
3. Flight and landing phases (1.1–1.6 s): The legs fully extended during flight (contact force ≈ 0 N), followed by controlled compression during landing to dissipate impact energy.

The PD controller effectively tracked the desired positions of the wheel axle within the base frame, with stiffness and damping coefficients set to $K_{p}=20$ and $K_{d}=1$, respectively. The virtual forces generated by the controller were mapped to the active leg motor torques using the Jacobian matrix ((18)), enabling dynamic leg actuation. The robot achieved a jumping height of approximately 0.1 m, as verified through manual measurement (using a tape measure) when state estimation became unreliable during the flight phase. It is worth noting that the PD gains were intentionally kept moderate, as the primary function of the leg in this context is to act as an active suspension system for navigating uneven terrain.

The jumping maneuver explicitly drives the system far from its linearized equilibrium. To quantify this, Table 1 reports the extreme values of the key states recorded during the jump depicted in Figure 9 and Figure 10.

It is critical to note that the LQR controller used was the same single-gain controller designed for the nominal upright position; no gain scheduling or online relinearization was activated during the jump. To assess whether the controller remained effective despite the large pitch angle, we monitored the quadratic cost term $x_{k}^{⊤}Qx_{k}$ relative to its value during steady-state balancing. This cost increased by a factor of 4.5 during landing but rapidly decayed. This indicates that the state entered a region where the linear model was not accurate, but the control law, derived from that model, still provided a stabilizing action that successfully recovered the robot.

### 4.2. Experiments of the Self-Balancing Wheelchair

The self-balancing wheelchair is driven by two DC servo motors, which are controlled by an Intel NUC computer through an EtherCAT bus. Each motor can generate a maximum torque of 70 Nm. An Xsens MTi 630 IMU sensor (Movella company, El Segundo, CA, USA) is mounted on the chair’s base, while a Sunrise M3715B 6-axis Force/Torque sensor (Sunrise Instruments company, Canton, MI, USA) is positioned between the chair and the axle for precise measurement. The base mass $m_{b}$ is 15.5 kg, while the mass of the wheel is 1.9 kg. The CoM height *l* is 0.128 m. The radius *r* of the wheel is 0.3 m. A Kalman filter is also employed to make optimal state estimation by merging the IMU and the wheel odometer.

The high-level control loop, encompassing state estimation, model updating, and LQR control law computation, runs at a deterministic 200 Hz. The data confirms that the worst-case execution time is well within the 5 ms available per cycle, leaving a sufficient computational margin.

#### 4.2.1. Wheelchair Experiments

**Figure 11:**
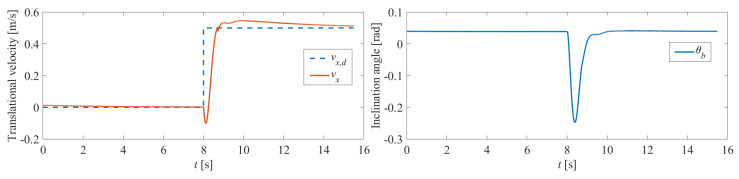
Velocity tracking experiment of the self-balancing wheelchair.

**Figure 12:**
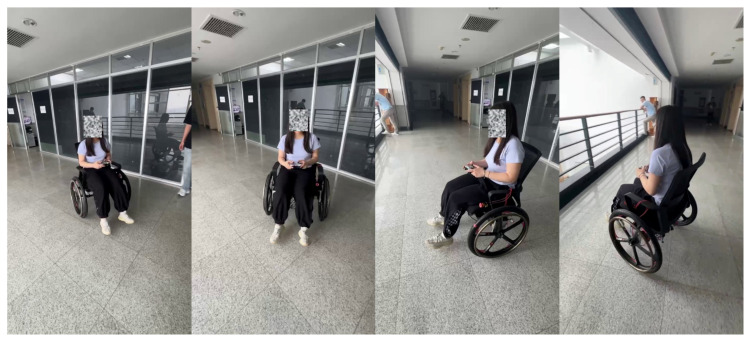
Snapshots of the self-balancing wheelchair carrying a person.

**Figure 13:**
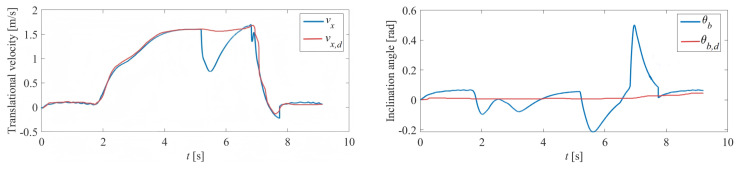
The recorded states as the wheelchair overcame a 5 cm height step. The robot came into contact with the step at 5 s.

The velocity tracking experiment was the first conducted to validate the performance of the proposed controller. Figure 11 illustrates the results obtained when the robot, carrying a person weighing 55.1 kg, was tested. The dynamic model was updated by the proposed model estimation algorithm using the force/torque sensor when the person was sitting down. The estimated mass is 56.12 kg, which is within a 2% relative error of the actual mass (55.1 kg). We deem this error margin reasonable given the potential for small shifts in the passenger’s posture and the inherent noise in the force/torque sensor readings during the brief static measurement period. The matrix Q in the LQR was kept at (20,000, 30, 500), while the matrix *R* in the LQR was kept at 1. The controller demonstrates strong performance in tracking step commands, while the inclination angle is rapidly regulated back to the equilibrium point, as shown in Figure 11. Figure 12 depicts real robot experiments where the user sent commands via a joystick. The LQR controller and the PD turning controller ($K_{p}=45$ and $K_{d}=1$) coordinated effectively while carrying the person. Figure 13 demonstrates the recorded velocity and inclination angle as the wheelchair overcame a 5 cm height step. The wheels made contact with the step at around the 5-s mark, after which the robot successfully surmounted the step and stabilized within 3 s. This experiment highlights the potential application of this human-carrying wheelchair as it demonstrates superior obstacle-crossing capabilities compared to traditional four-wheeled wheelchairs.

#### 4.2.2. Safety System and Experimental Protocol

The operation of a self-balancing wheelchair carrying a human passenger necessitates a rigorous safety-first approach. This subsection documents the safety interlocking, comfort metrics, and experimental criteria employed.

A defense-in-depth strategy was implemented to ensure passenger safety:

1. Software Safety Limits: The control software incorporated hard limits on critical states. If the body pitch angle $|\theta_{b}|$ exceeded 15° or the translational velocity exceeded 1.5m/s, the controller would cut power to the motors and initiate a controlled braking procedure.
2. Watchdog Timer: A dedicated hardware watchdog timer was used to monitor the high-level control loop. If a computation hang or overrun prevented the loop from being complete within its 5ms deadline, the watchdog would trigger a system halt.
3. Emergency Stop (E-Stop): A latching, physical E-stop button was installed and made readily accessible to both the passenger and an external operator. Engaging the E-stop cut all motor power independently of the main computer.
4. Safety Assistant: During all experiments, the wheelchair operated within a controlled laboratory space. The passenger was protected by other people in case of tip-over.

### 4.3. Ablation Studies and Quantitative Analysis

To rigorously evaluate the contribution of each component in the proposed control framework, a series of ablation studies and quantitative tests were conducted on the self-balancing wheelchair platform. All quantitative results are reported as the mean ± standard deviation across N = 5 trials for each condition.

#### 4.3.1. Benefits of Online Model Update

We first isolated the benefits of the online model-updating algorithm. The controller was tested under three passenger conditions: no passenger, a 55 kg passenger, and an 85 kg passenger. We compared the proposed online LQR (model parameters updated via F/T sensor) against a fixed LQR (parameters fixed for the no-passenger condition).

As shown in Table 2, the performance of the fixed LQR degraded severely with increasing passenger mass and became unstable with the 85 kg passenger. In contrast, the online LQR maintained consistently low tracking error and stability across all conditions, demonstrating its critical role in handling large model variations.

#### 4.3.2. Benefits of Pitch Bias Compensation

We further isolated the effect of the equilibrium pitch bias compensation $\theta_{b,d}$. Using the online LQR, we tested with a 55 kg passenger with a known CoM horizontal offset of 8 cm. We compared performance with and without the force/torque-sensor-based $\theta_{b,d}$ to update Equation (25).

The results in Table 3 show that without pitch bias compensation, the system settles at an incorrect equilibrium with a large steady-state pitch error, requiring constant control effort to maintain this offset posture. Our compensation method successfully corrects the equilibrium, bringing the steady-state pitch to zero and significantly reducing the control effort.

## 5. Conclusions

This paper presented a unified control framework to address the fundamental challenge of model variations in two distinct self-balancing robot platforms: a wheel-legged robot and a human-carrying wheelchair. The core of our approach lies in the choice of an online model-updating LQR controller as the universal balancing core. This design was strategically selected because the LQR provides a principled and computationally tractable optimal control solution, while the real-time model update mechanism directly compensates for the primary source of uncertainty—changes in mass and center of mass. This contrasts methods that treat model variations as unstructured disturbances, enabling our controller to proactively reconfigure for new operating conditions. The wheel-legged robot achieved a 0.1 m jump height and maintained reasonable velocity error, while the wheelchair overcame a 5 cm step with a 3 s settling time while carrying a 55 kg passenger. The model updating algorithm estimated passenger mass and CoM with high accuracy, enabling dynamic adjustments. These results validate the framework’s robustness and potential for applications in personal mobility and assistive robotics, with future work focusing on optimization for complex environments and enhanced sensor integration.

Future work will focus on several key areas. First, we plan to conduct comprehensive testing in more complex environments. Furthermore, a systematic quantitative comparison of the proposed unified framework against state-of-the-art alternative strategies, such as nonlinear MPC and gain-scheduled LQR, will be undertaken to rigorously benchmark its performance, advantages, and limitations. Also, reinforcement learning control methods, particularly the Actor–Critic reinforcement learning method, may be promising alternative algorithms for the model variation problem.

## Figures and Tables

**Table 1:** Maximum state deviations during the jumping experiment.

State Variable	Maximum Value	Phase of Occurrence
Body Pitch Angle $\theta_{b}$	16.8°	Landing Impact (at 1.4 s)
Body Pitch Rate ${\dot{\theta }}_{b}$	142.5°/s	Landing Impact (at 1.4 s)
Wheel Contact Force	38 N	Take-off (at 1.0 s)

**Table 2:** Ablation study: online vs. fixed LQR.

Passenger Mass	Controller	RMSE of $v_{x}$ (m/s)	Success Rate
0 kg	Fixed LQR	0.032 ± 0.005	5/5
0 kg	Online LQR	0.029 ± 0.004	5/5
55 kg	Fixed LQR	0.158 ± 0.021	5/5
55 kg	Online LQR	0.035 ± 0.006	5/5
85 kg	Fixed LQR	Unstable (Fell)	0/5
85 kg	Online LQR	0.041 ± 0.007	5/5

**Table 3:** Ablation study: with vs. without pitch bias compensation.

Condition	Steady-State Pitch Error (°)	RMS of τ (Nm)
Without $\theta_{b,d}$ compensation	−4.8 ± 0.3	5.2 ± 0.6
With $\theta_{b,d}$ compensation	−0.2 ± 0.1	1.8 ± 0.3

## Data Availability

Data is available if required by email.
